# Comparison of two surgical approaches for displaced intra-articular calcaneal fractures: sinus tarsi versus extensile lateral approach

**DOI:** 10.1186/s12891-015-0519-0

**Published:** 2015-03-19

**Authors:** Je-Hyoung Yeo, Hyun-Jong Cho, Keun-Bae Lee

**Affiliations:** Department of Orthopedic Surgery, Chonnam National University Medical School and Hospital, 42 Jebongro, Donggu, Gwangju 501-757 Republic of Korea

**Keywords:** Calcaneus, Intra-articular fracture, Sinus tarsi approach, Extensile lateral approach

## Abstract

**Background:**

Two common surgical approaches included the sinus tarsi and extensile lateral are used for displaced intra-articular calcaneal fractures. However, few studies have compared outcome of treated by the two approaches. The purpose of this study was to compare the outcome between these two approaches for Sanders type-II and type-III fractures.

**Methods:**

This retrospective cohort study was performed from 2004 to 2011. Open reduction and internal fixation using the sinus tarsi and extensile lateral approach was studied in 100 cases (40 sinus tarsi and 60 extensile lateral) with displaced intra-articular calcaneal fractures. All patients were evaluated both clinically and radiologically.

**Results:**

Median Böhler and Gissane angle were improved to 26.5 degree (4.6 to 45), 115.5 degree (101.2 to 127.4) in the sinus tarsi group and 25.3 degree (3.7 to 44.6), 119.0 degree (73.5 to 145.6) in extensile lateral group at the final follow-up, respectively. Median calcaneal height, length, and width in the sinus tarsi and extensile lateral groups showed improvement to 45.1 mm (23.2 to 54.1), 75.9 mm (64.9 to 90.3), 37.6 mm (29.2 to 53.9) and 46.5 mm (32.7 to 59.5), 76.1 mm (67.3 to 97.9), 39.3 mm (29.2 to 47.8) at the final follow-up, respectively. Median AOFAS score was checked to 90 points (76 to 94) in the sinus tarsi group and 86 points (76 to 94) in the extensile lateral group at the final follow-up. No significant differences in clinical and radiologic outcomes were observed between the two groups. However, wound complication rate (13.3%) in the extensile lateral group was significantly higher compared to the sinus tarsi group (*p*-value = 0.022).

**Conclusions:**

The final clinical and radiographic outcomes between the two approaches for Sanders type-II and type-III intra-articular calcaneal fractures were comparable and equally successful. The selective sinus tarsi approach appears to be an effective and reliable method for the treatment of Sanders type-II and type-III fractures.

## Background

Displaced intra-articular calcaneal fractures (DIACF) have been recognized as a source of significant disability and known to accompany by complication after the treatment period. Therefore, DIACF are one of the most difficult articular fractures to treat and clinical outcomes do not satisfy surgeons and patients [1-3]. Although the best option for treating DIACF can be operative or non-operative, controversy remains as to the optimal treatment of the injury, because both operative and non-operative treatments have advantages and disadvantages [4-7]. However, many surgeons still have a choice operation for treatment of DIACF. Operative treatment for DIACF consists of open reduction internal fixation, the Essex–Lopresti reduction maneuver, and primary arthrodesis [8,9].

The goal of operative treatment is to acquire anatomic reduction of the articular surface, restore the subtalar joint and normal width of the calcaneus, and to maintain this reduction with stable fixation [3,9-11]. Several open surgical techniques such as medical, lateral, combined lateral and medial, and posterior approaches have been described, of which the extensile lateral approach has gained wide popularity for treatment of DIACF [9,12,13].

The extensile lateral approach provides excellent visualization of the fracture site, allowing access to manipulate and rigidly fix the injury and directly reduce the displaced fracture fragment [3,12]. Despite meticulous attention to soft tissue management, a fairly high complication rate has been seen with this approach, including wound healing complications, deep infections, sural nerve injuries, and subtalar arthritis [14-16].

Because of these problems, there has been renewed interest in small incision surgery for calcaneal fractures [17]. A small incision has been described with various modifications, such as the sinus tarsi approach [17,18]. These techniques all attempt to minimize soft tissue trauma, thereby minimizing the risk of operative complications, while still allowing good fracture reduction. However, these approaches also have some problems such as technical difficulties, poor visualization of the fracture site, and difficulty with manipulation [19].

Several studies have described the clinical outcomes associated with the extensile lateral and sinus tarsi approached [16-18]. However, few studies have compared results of operative fixation of DIACF treated with the sinus tarsi approach versus those treated with the traditional extensile lateral approach. One study reported that the clinical results are similar between DIACF treated by the extensile lateral approach and those treated by a minimally invasive approach. However, the minimally invasive approach has a significantly lower incidence of wound complications and secondary intervention [19].

We hypothesized that the outcomes between Sanders type-II and type-III fractures treated with the sinus tarsi and extensile lateral approaches are comparable and successful. Accordingly, the purpose of the present study was to compare the clinical and radiological outcomes between the two surgical approaches for Sanders type-II and type-III fractures.

## Methods

### Patient populations

A total of 130 cases underwent open reduction and internal fixation for treatment of DIACF from September 2004 to February 2011. All procedures were performed by a single surgeon. Inclusion criteria included patients over the age of 17 years, fracture classified as Sanders type-II and type-III, closed fractures, and fractures treated with operative management via the sinus tarsi or extensile lateral approach. A total of 30 cases were excluded for the following reasons were less than 17 years of age (four cases), were classified as Sanders types-I, type-IV, and open fracture (18 cases), and those with extra-articular fractures (eight cases). The remaining 100 cases were enrolled in this study. The 100 cases were divided into two group; a sinus tarsi approach group (40 cases, STA group) and an extensile lateral approach group (60 cases, ELA group). Both approaches were used contemporaneously based on surgeon preference. This retrospective cohort study was approved by the institutional review board of Chonnam National University Hospital and verbal informed consent for participation in the study was obtained from all patients, including permission to access patients’ records and to publish individual clinical details.

A meticulous clinical chart and radiographic review were performed for all patients. Demographic data were collected for all patients including age, sex, tobacco use, presence or absence of diabetes, injury side, time to surgery, operation time, and follow-up duration. The Sanders classification was determined based on computed tomography (CT) scans. Demographic data are shown in Table 1. The STA group consisted of 25 men and 15 women (median age, 46 years). The median follow-up duration was 46 months (26 to 100). The ELA group consisted of 38 men and 22 women (median age, 42 years). The median follow-up duration was 57 months (36 to 96). The STA group consisted of 25 patients in Sanders type-II (62.5%) and 15 in Sanders type-III (37.5%) versus the ELA group with 37 Sanders type-II (61.6%) and 23 Sanders type-III (38.4%).

**Table 1:** **Demographic data between the sinus tarsi and extensile lateral approach groups**

	**Sinus tarsi (N = 40)**	**Extensile lateral (N = 60)**
Sex, male/female, n	25/15	38/22
Age, y	46 (20 to 65)	42 (17 to 64)
Tobacco, n (%)	8 (20%)	13 (21.6%)
DM, n (%)	1 (2.5%)	2 (3.3%)
Side of injury, Rt/Lt, n	22/18	36/24
Time to surgery, d	7 (0 to 14)	7.5 (0 to 16)
Operation time, min	61.7 (40 to 75)	76.3 (65 to 95)
Sanders classification, n (%)		
IIA; IIB; IIC	15;8;2 (62.5%)	13;18;6 (61.6%)
IIIAB; IIIAC; IIIBC	10;5;0 (37.5%)	11;8;4 (38.4%)
Follow-up duration, m	46 (26 to 100)	57 (36 to 96)

Values are expressed as median (range) unless otherwise indicated.

### Radiological evaluations

CT scans were obtained to confirm Sanders classification preoperatively and were used to detect the posterior facet and fracture to assist in preoperative planning. Classification of DIACF according to Sanders [11] is based on coronal computed tomographic scans.

The plain radiographic evaluations included anteroposterior and lateral view of the calcaneus, the calcaneal tangential view, and Broden’s view in all patients. The Gissane and Böhler angles, calcaneal height, length and width were also checked. The radiologic evaluations were performed preoperatively and postoperatively at one month, three, six and twelve months and then annually thereafter. To avoid potential bias, plain radiographs and preoperative CT scans were evaluated by two independent observers who were not involved in the surgical treatment of the patients and who were blinded to the intention of this study.

### Clinical evaluations

American Orthopedic Foot and Ankle Society (AOFAS) ankle-hindfootscale scores, visual analog scale (VAS) pain scores, and the foot function index (FFI) were used to evaluate clinical outcomes. Results were obtained postoperatively at one month, three, six and twelve months and then annually thereafter. To avoid examiner bias, clinical scoring were evaluated by two independent observers who were not involved in the surgical treatment of the patients and who were blinded to the radiologic finding.

The 100-point AOFAS scoring system considers a score of ≥ 90 points as excellent, 80–89 points as good, 70–79 points as fair, and a score of ≤ 69 points as poor [20]. The VAS pain score was used to measure the amount of pain patients felt between 0 and 10 points and contained word descriptors. The FFI provides information on how foot pain affects the patient’s ability to manage everyday life; points are allocated as follows: pain, disability, and activity limitation subscale. Total score range from 0 to 100. Higher scores indicate greater impairment.

The rates of wound healing complications, deep infections, sural nerve injuries, peroneal tendinitis, and subtalar stiffness were evaluated.

### Operative techniques

The initial treatment with ice and elevation should be continued until soft tissue swelling subsides and skin wrinkles are apparent.

#### Sinus tarsi approach

**Figure 1:**
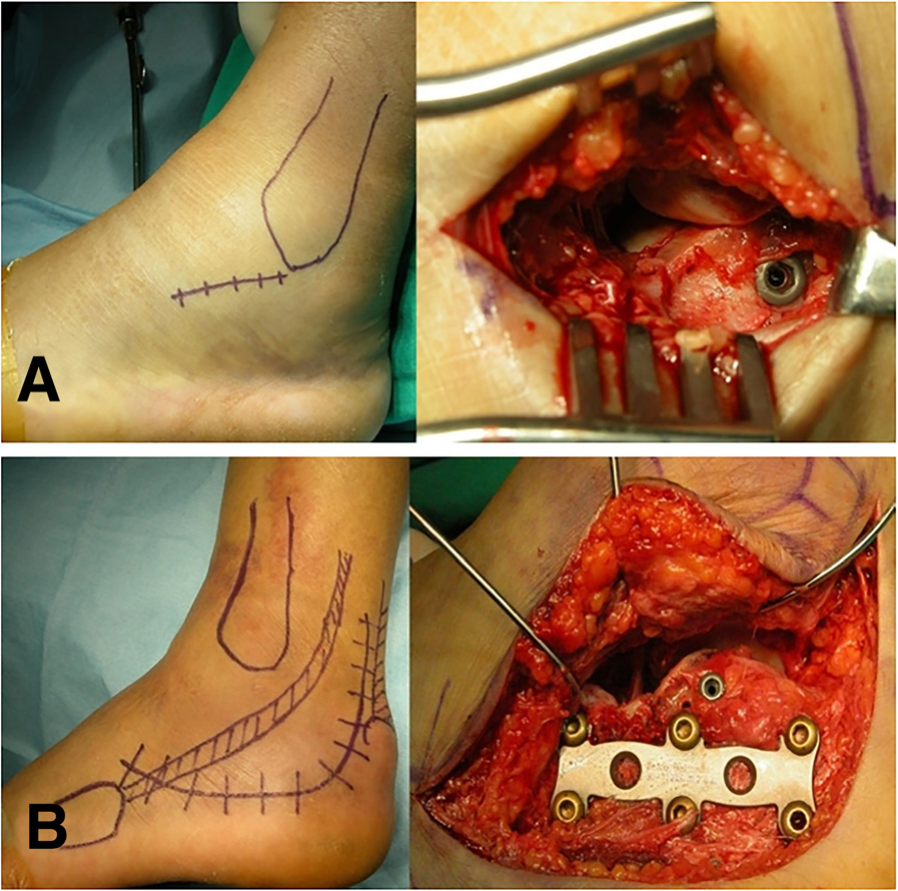
**Photograph showing skin incision line and surgical approach. (A)** Sinus tarsi approach (STA) **(B)** Extensile lateral approach (ELA).

Approach was performed by placing the patient in either lateral decubitus or semilateral position with the use of a beanbag on a translucent table under general anesthesia using a thigh tourniquet. We used a straight incision on the lateral side of the foot just distal to the tip of the fibula and roughly horizontal to the sole of the foot. The incision started approximately one cm posterior to the fibula and continued distally for about 3–4 cm. The extensor digitorum brevis was retracted cephalad, and the peroneal tendon was retracted inferiorly for allowing exposure of the sinus tarsi. A Schanz pin was placed in the lateral aspect of the calcaneal tuberosity fragment to restore calcaneal length and height and to correct the varus deformity. Once the fractures were reduced, temporary K-wire fixation was used to hold the fragments in place. Then, a 3.5 mm cannulated lag screws (DupeySynthes, West Chester, PA, USA) were passed across the fracture site from lateral to medial to hold the sustentacular bone. A 6.5 mm cannulated screws (DupeySynthes, West Chester, PA, USA) were then placed percutaneously from the posterior aspect of the calcaneus. Once rigid fixation was achieved, the incision sites were closed in a layer by layer fashion (Figure 1A).

#### Extensile lateral approach

Approach was performed by placing the patient in either the lateral decubitus or semilateral positions with the use of a beanbag on a translucent table under general anesthesia using a thigh tourniquet. After exsanguination of the lower extremity, the calcaneus is approached through an L-shape incision. The incision begins laterally 3–4 cm superior to the calcaneal tuberosity and 1–2 cm anterior to the heel cord. The incision was extended distally and continued retrofibularly to the junction of the dorsal and plantar skin, where a smooth curve was made, curving the incision anteriorly toward the calcaneocuboid joint and the fifth metatarsal base. This approach minimizes sequelae of peroneal tendinitis and devascularization of the anterior skin flap and preserves the sural nerve. The fracture line at the level of the Gissane angle was identified, and the thin lateral wall was retracted inferiorly to expose the articular fracture fragments. Attention was turned to restoring the height, width, and length of the calcaneus, with was accomplished by complete reduction of articular fragments. Then, 3.5 mm cannulated lag screws were placed from the lateral cortex toward the sustentaculum [21]. An H-plate (DupeySynthes, West Chester, PA, USA) or calcaneal locking plate (DupeySynthes, West Chester, PA, USA) was applied for the calcaneus to stabilize the posterior facet, the anterior process, and the posterior tuberosity. Once rigid fixation was achieved, the wound was closed in a layer by layer fashion (Figure 1B).

The postoperative management regimen was the same for both groups. The patient was placed in a well-padded short leg splint and a bulky dressing with the leg elevated for 2–3 days. Once the wound was healed after two or three weeks postoperatively, active range of motion of the ankle and subtalar joint was started. Protection is provided by the use of a removable posterior splint. Weight bearing is instituted at 10 to 12 weeks, extensive physical therapy is started.

### Statistical analysis

The independent t-test was used to determine the significances of inter-group differences in age and follow-up duration. The Mann–Whitney *U*-test used to determine the significance of inter-group differences for the clinical and radiographic outcomes. Pearson’s chi-square test was performed to determine the significance of intergroup differences for the prevalence of complications. A *p*-value < 0.05 was considered significant and the statistical analysis was independently performed by a statistician.

## Results

### Radiographic outcomes

The radiographic results are summarized in Table 2. All fractures achieved successful union without any adverse events such as inadequate reduction, non-union, malunion, and fixation failure. Median Böhler angle was improved to 26.5 degree (4.6 to 45) in STA group and 25.3 degree (3.7 to 44.6) in ELA group at the final follow up. Median Gissane angle was improved to 115.5 degree (101.2 to 127.4) in STA group and 119.0 degree (73.5 to 145.6) in ELA group at the final follow up.

**Table 2:** **Radiographic outcomes between the sinus tarsi and extensile lateral approach groups**

**Outcomes**	**Sinus tarsi**		**Extensile lateral**		***p-*** **value†**
	**Preop**	**Final follow-up**	**Preop**	**Final follow-up**	
Böhler angle, degree	17.0 (0.1 to 35)	26.5 (4.6 to 45)	16.6 (0.3 to 46.2)	25.3 (3.7 to 44.6)	0.409
Gissane angle, degree	120.1 (95.1 to 148.6)	115.5 (101.2 to 127.4)	121.8 (81 to 141.7)	119.0 (73.5 to 145.6)	0.424
Height, mm	42.2 (23.2 to 54.1)	45.1 (23.2 to 54.1)	41.3 (27.5 to 49.5)	46.5 (32.7 to 59.5)	0.371
Length, mm	76.3 (46.7 to 92.0)	75.9 (64.9 to 90.3)	75.3 (64.4 to 96.9)	76.1(67.3 to 97.9)	0.423
Width, mm	40.0(29.6 to 54.2)	37.6(29.2 to 53.9)	39.2(32.5 to 64.9)	39.3(29.2 to 47.8)	0.419

Values are expressed as median (range).

† Mann–Whitney *U*-test.

**Figure 2:**
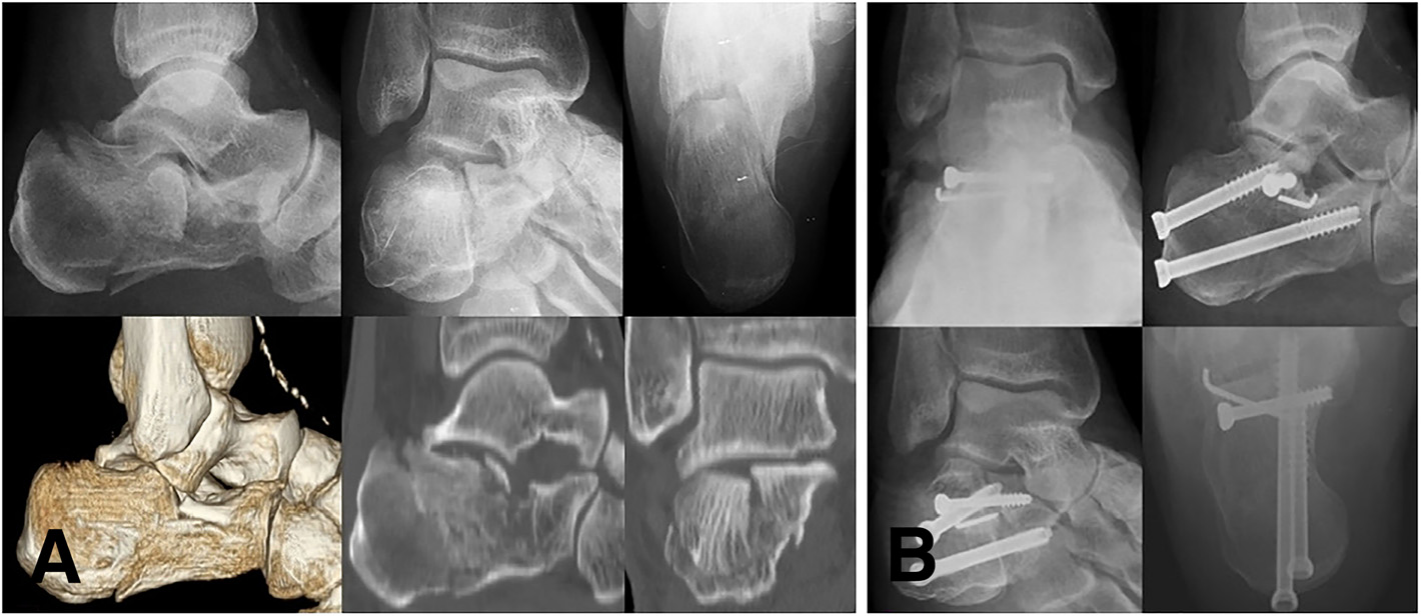
**Radiologic evaluations of a 55 year old male patient with Sanders type-II intra-articular calcaneal fracture treated with open reduction and internal fixation using the sinus tarsi approach. (A)** Preoperative plain radiographs and CT scans **(B)** Postoperative plain radiographs.

**Figure 3:**
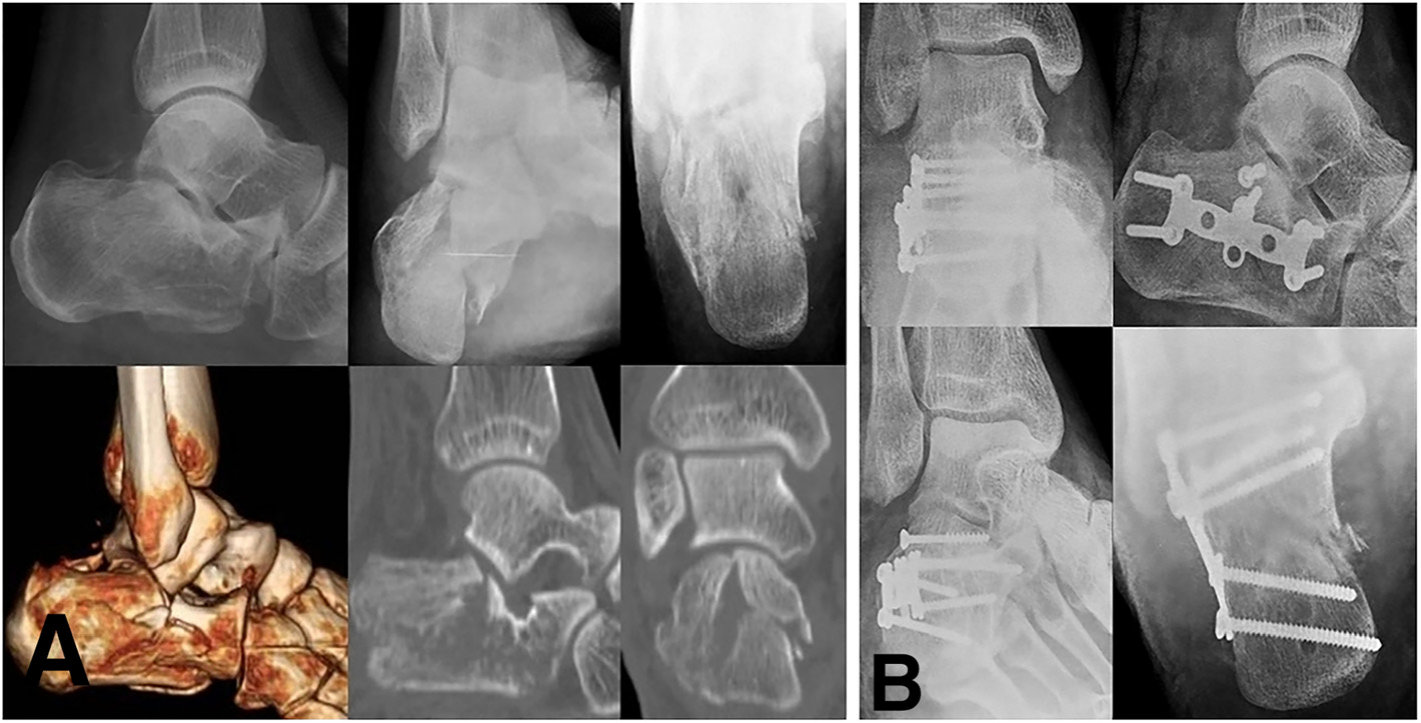
**Radiologic evaluations of 52 year old male patient with Sanders type-II intra-articular calcaneal fracture treated with open reduction and internal fixation using the extensile lateral approach. (A)** Preoperative plain radiographs and CT scans **(B)** Postoperative plain radiographs.

Median calcaneal height, length, width was improved to 45.1 mm (23.2 to 54.1), 75.9 mm (64.9 to 90.3), 37.6 mm (29.2 to 53.9) in the STA group and 46.5 mm (32.7 to 59.5), 76.1 mm (67.3 to 97.9), 39.3 mm (29.2 to 47.8) in the ELA group at the final follow up, respectively. Radiographic outcomes were not significantly different between the two groups at the final follow up (*p*-value > 0.05) (Figures 2 and 3).

### Clinical outcomes

Median AOFAS scores were checked to 90 points (76 to 94) in the STA group and 86 points (76 to 94) in the ELA group at the final follow up. The overall results according to AOFAS scores were excellent in 24 (60.0%) patients; good in 15 (37.5%), fair in one (2.5%) in the STA group and were excellent in 30 (50%) patients; good in 28 (46.6%), and fair in two (3.4%) in the ELA group; yielding a 96.6% and 97.4% excellent or good rating in both groups. Median VAS scores were checked to 2 points (1 to 5) in the STA and 2 points (1 to 5) in the ELA group at the final follow-up. Median FFI scores were checked to 9.6 points (7.8 to 13.5) in the STA group and 9.6 points (7.8 to 15.2) in the ELA group at the final follow-up. No significant differences were observed between the groups for these scores (*p*-value > 0.05).

### Complications

The complications are summarized in Table 3. Eight cases (13.3%) of wound healing complications occurred in the ELA group. And two (5%) wound healing complications including wound dehiscence and prolonged drainage were observed in the STA group. However, none required operative treatment. The presence or absence of residual numbness in the sural nerve distribution was determined from clinical review. Four (6.6%) of 60 patients in the ELA group complained of sural nerve symptoms. However, no patient required exploration or sural neuroma excision. Two (5%) of 40 patients in the STA group complained of sural nerve symptoms but no patient required intervention. One case of peroneal tendinitis was observed in the ELA group. However, no patient required operative treatment. No peroneal tendinitis was observed in the STA group. Five (8.3%) of 60 patients in the ELA group complained of subtalar stiffness, and one required exploration and arthroscopic subtalar release. The indications of arthroscopic subtalar release were isolated subtalar stiffness with pain on weight bearing which was aggravated by walking on uneven ground; articular incongruity less than 2 mm at the posterior facet of the subtalar joint on CT scans; and failure to respond to conservative treatment. The procedure was debrided of fibrous tissue in the sinus tarsi and lateral gutter and released the posterior talocalcaneal facet. Arthroscopic subtalar release was performed before posttraumatic arthrosis occur [22]. Four patients reduced their pain by doing subtalar circle motion exercises. Three (7.5%) of 40 patients in the STA group complained of subtalar stiffness, and one required exploration and surgical release. Two patients reduced their pain by subtalar circle motion exercises.

**Table 3:** **Complications between the sinus tarsi and extensile lateral approach groups**

	**Sinus tarsi**	**Extensile lateral**	***p-*** **value†**
Nonunion	0	0	0
Wound complication	2 (5%)	8 (13.3%)	0.022
Deep infection	0	0	0
Sural nerve injury	2 (5%)	4 (6.6%)	0.430
Peroneal tendinitis	0	1 (1.6%)	0.444
Subtalar stiffness	3 (7.5%)	5 (8.3%)	0.458
Reoperation	0	0	0

Values are expressed as number (percentage).

†Pearson’s chi-square test.

## Discussion

This study is to compare the outcomes between STA group and ELA group for DIACF. The major finding of the study was that the final clinical and radiologic outcomes between the two groups for Sanders type-II and type-III fractures were comparable and equally successful. The ELA group had a higher complication rate, particularly wound healing complications, than that in the STA group.

The most popular approach for the open reduction and internal fixation of calcaneal fracture has been the ELA [9,12,13,23]. This approach provides excellent visualization and allows access to manipulate and rigidly fix the injury with direct reduction [13,17], but wound complications arise with this approach, and complication rates vary from 11–25% [14-16,24]. Weber et al. [25] reported that the rate of injuries to the sural nerve was 7.7% in their patients treated via the ELA. In our study, percentage of complications similar to the literature were identified.

Because of these problems with the ELA, there has been renewed interest to develop alternative techniques to manage intra-articular calcaneal fractures and minimize soft tissue complications [17,18,26,27]. Many techniques have been described over the last 10 years [26,28-30], including percutaneous fixation, arthroscopic assisted, external fixation, trans-articular, and small medial, posterior, lateral or a combined incision technique [17,26-30]. The less invasive approach has been described such as the STA [17,18]. These techniques all attempt to minimize the risk of operative complications, while still allowing good fracture reduction. We focused here on our studies describing the STA.

Holmes [18] described the minimally invasive STA for displaced intra-articular calcaneal fractures. The author reported that the STA provides for adequate exposure to accomplish successful reduction and fixation without any wound dehiscence, osteomyelitis, or wound infection over an 18-years period.

Hospodar et al. [31] evaluated 16 consecutive cases using the minimally invasive STA. No major wound complications were reported. The posterior facet joint was successfully reduced to < 2 mm of displacement in 14 patients, and 12 patients were back to work by 6 months postoperatively. In STA group of this study, we achieved successful bony union and had only two minor wound complications.

In terms of outcome comparison of these two approaches for DIACF, Kline et al. [19] reported on a retrospective trial of 112 displaced intra-articular calcaneal fractures treated with the ELA (79 cases) or STA (33 cases). The author described that the clinical results were similar between calcaneal fractures treated with the two approaches. However, the STA had a significantly lower incidence of wound complications and secondary surgery. They concluded that the minimally invasive approach was a valuable method for treating intra-articular calcaneal fractures.

There are some limitations in this study. First, our average follow-up period was approximately four years, mid-term follow-up period. Although this would have certainly revealed any early complications, it would did not capture all patients who developed symptomatic subtalar arthritis in the future. However, the present study can provide useful information on the outcome comparison between the two approaches for treating the DIACF. Second, largely inherent in its retrospective manner. As there was no randomization and the decision for the type of approach was at the discretion of the surgeon and was not based on an established protocol. Third, it is insufficient to evaluate outcomes according to the extent of comminution and degree of displacement, and reduction state of fracture were not mentioned due to a lack of postoperative CT scan.

## Conclusions

The final clinical and radiographic results of the STA were comparable and successful to the ELA. Moreover, the STA had a lower rate of wound complications than that of the ELA. We believe that the STA has become an effective and reliable method to treat Sanders type-II and type-III DIACF.

## Abbreviations

DIACF: Displaced intra-articular calcaneal fracture
STA: Sinus tarsi approach
ELA: Extensile lateral approach
AOFAS: American Orthopedic Foot and Ankle Society
VAS: Visual analog scale
FFI: Foot function index
